# Veterinary nanomedicine: Pros and cons

**DOI:** 10.1002/vms3.1050

**Published:** 2022-12-29

**Authors:** Fariba Jafary, Shima Motamedi, Isaac Karimi

**Affiliations:** ^1^ Department of Biochemistry Najafabad Branch Islamic Azad University Najafabad Iran; ^2^ Graduate of Doctor of Veterinary Medicine School of Veterinary Medicine Razi University Kermanshah Iran; ^3^ Department of Biology Faculty of Science Razi University Kermanshah Iran

**Keywords:** animal production, food industry, health care production, nanotechnology, nanomedicine, pharmacology, veterinary medicine

## Abstract

In recent years, nanotechnology has improved life with continuous growth in different fields. Nanoparticles can be employed in industry, imaging, engineering, and various biomedical filed because of their special physicochemical properties like rapid, effective, highly specific solutions, higher stability, biodegradability, biocompatibility, and cost. In this line, veterinary medicine has been influenced by nanotechnology in prevention, diagnosis, and treatment of diseases, cancer therapy, immunization, vaccine production, drug delivery, and health besides to related issues of animal production, maintenance, and welfare. The other important point is the interwoven linkage between animals and humans whether as a food source or as a companionship. Inorganic nanoparticles, polymeric, solid lipid, liposomal, nanocrystal, nanotubes, nanoemulsions, micelles, mesoporous silica nanoparticles, and dendrimers are kinds of nanoparticles that can be used widely. In this review, the impacts of nanotechnology on veterinary medicine have been summarized, criticized, and acknowledged as “veterinary nanomedicine” discipline.

## Graphical abstract

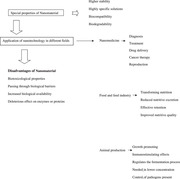

## 1 INTRODUCTION

### 1.1 Nanotechnology

There are several nanosized compounds ranging from deoxyribonucleic acid (DNA; 2.5 nm), red blood cells (RBC;7 nm), protein (5–50 nm), and virus (75–100 nm) as compared with human hair (80,000 nm) (Uniyal et al., 2017; Zampoli Troncarelli et al., 2013). The nanocompounds have been produced for several years on earth planet by natural phenomena arranged from forest burning and volcanoes to photochemical reactions (Uniyal et al., 2017). Indeed, humans always tried to discover new and better solutions for easing their life regards to present natural models. The seeded nanotechnology was one of these fields that was founded for the first time by Richard Feynman, the well‐known physicist in 1959 (Gopi et al., 2017); however, the first use of nanotechnology term back in 1974 (Zampoli Troncarelli et al., 2013). In a general and simple view based on its etymology, nanotechnology is a technology of nanometer materials (Ashok & Canetta, 2020; Scott, 2005; Zampoli Troncarelli et al., 2013) while, *nano*, the Greek word, means “dwarf” (Gopi et al., 2017). This technology is based on engineering, manipulation, measurement, and organizing of material in 1–100 nm or sometimes <1 nm scale (DeLouise, 2012; Hill & Li, 2017; Mohanty et al., 2014; Sauer, 2009; Uniyal et al., 2017; Zampoli Troncarelli et al., 2013). This interdisciplinary field has various applications in environment, paint design, engineering, electronic devices, food and feed, biology, weaving, agriculture, sporting goods, cosmetics, pharmacology, medicine, biotechnology, and veterinary medicine (Aschberger et al., 2015; Cubadda, 2007; Martirosyan & Schneider, 2014; Mohanty et al., 2014; Pati et al., 2009; Sauer, 2009; Scott, 2005; Swain et al., 2015; Uniyal et al., 2017). The reason for these various and broad applications is economic return and producing more efficient and new or even reproducing the present materials more effectively and economically via combining various fields of research. Furthermore, environmental pollutions and global warming which are caused by nowadays consumed materials and methods require more effective and less dangerous solutions and alternatives.

There are different nanomaterial (NM) classifications since variable nanotechnological usages need a wide range of materials with variable shapes and unique properties. The aforementioned properties of nanoparticles (NP) besides their unique surface phenomenon, tunable absorption, and emission properties make them candidates to be used in carrying therapeutics, gene‐therapy for diagnostics, treating diseases, especially cancer and central nervous system‐related diseases besides being used as magnetic resonance imaging (MRI) or bio‐molecular imaging contrast agents (Khan et al., 2019). Of course, these engineered particles have been used for vaccine carriers and inhalation delivery of poor‐soluble drugs such as corticosteroids, hormones, and antifungal agents (Khan et al., 2019; Pandit et al., 2022). Their present shapes include nanotubes (NT), fullerenes, liposomes, dendrimers, polymeric micelles, polymeric nanospheres, nanofibres (NF), nanomembranes, nanocrystals, nanoshells, quantum dots (QD), stabilized solid lipids (SLN), nanoemulsions, nanoclays, and NP (Carvalhoa et al., 2020; Gopi et al., 2017; Kumar, 2015; Mohanty et al., 2014; Omanovic‐Miklicanin & Maksimovic, 2016) [Table 1, Scott, 2005; Zampoli Troncarelli et al., 2013].

**TABLE 1:** The classification of nanoparticles (NP) based on their physicochemical properties

Nanoparticle	Characteristics	Applications
Polymeric NP (nanospheres and nanocapsules)	Non‐toxic, biodegradable, no immunological reaction, thermolabile, biocompatible	Food‐drug therapeutics, tissue engineering, gene‐drug delivery, gene therapy for tumors
Micelles	A mixture of the amphiphilic polymers, hydrophilic portion, hydrophobic portion in various sizes	Drug delivery, antileishmanial activity, treatment of bovine mastitis
Liposomes	Possibility of working with hydrophilic and lipophilic drugs, ease of modulation of surface charge and size, functionalization capacity	Drug‐gene delivery, antivirals effect, immunoprotective capacity, vaccine treatment, treatment of infections, carcinogenic activity, anesthetic activity, oncotherapy
Dendrimers	Nanoscale sized, controllable size‐mass, uniform structure, good water‐solubility, optimized biocompatibility‐biodistribution, low polydispersity, structural reproduced, and no or low immunogenicity	Anti‐inflammatory, antibacterial, anticancer, gene delivery, drug delivery, photodynamic therapy, encapsulation, disease diagnosing, and industry fields
Nanoemulsions (NE)	Kinetically stable and thermodynamically biocompatible, and biodegradable	Drug delivery, cancer chemotherapy, antiparasitic effect, TEM images, photodynamic therapy
Solid lipid NPs (SLN)	Biocompatible, biodegradable, and stability	Antiparasitic activity, antileishmanial activity, vaccine adjuvant, drug delivery, optimize the immune response
Mesoporous silica NPs (MSN)	Kinetics and charge control, facilitated drug dissolution, and large pore volume	Gene and drug delivery, anti‐tumor effect and cancer therapy, antimicrobial activity
Metallic NPs	Safe, eco‐friendly, easy to make, rational cost, short synthesis time, and permeability	Biosensors, immunosensor, bioimaging, gene delivery, hyperthermia, cell labeling and drug delivery, antimicrobial and antiviral activity, bacterial infection treatment, anticancer therapy
Quantum dots (QD)	High photostability, resistance to photobleaching	Ideal for use in imaging purposes, fluorescent probes, highly sensitive low‐cost biosensors

There are diverse methods for the production and preparation of NMs and this list is growing. These methods include gas‐phase synthesis, emulsion cross‐linking, laser ablation, high energy ball mill, precipitation, spray dying, emulsion droplet coalescence, ionic gelation, convergent, divergent, reverse micelle, and eco‐friendly synthesis (Abbasi et al., 2014; Gopi et al., 2017; Rajput, 2015; Swain et al., 2015).

In eco‐friendly synthesis, biologic synthesis, biological micro‐organisms, plants, or fungi are used to make NP, especially metal ones. The NPs which have been synthesized via this method include gold (Au), silver (Ag), cadmium (Cd), selenium (Se), palladium (Pd), barium titanate (BaTiO3), titanium (Ti), and manganese (Mn) and there are many advantages in using these NPs such as being safe, being eco‐friendly, easy to make, rational cost, decreasing synthesis time, increasing permeability (Gopi et al., 2017; Swain et al., 2015). Depending on the plant source used in the synthesis process, the NP color will be different (Gopi et al., 2017).

The general NP characterization is determined by particle to mean size and diameter, distribution, morphology, and surface charge. After the synthesis of NPs, some techniques such as Scanning Probe Microscopy (SPM) techniques, Atomic Force Microscopy (AFM), Chemical Force Microscopy (CFM), Fluidic Force Microscopy (Fluid FM), Near‐field Scanning Optical Microscopy (NSOM), SNOM Raman spectroscopy (RS), Modulated Raman spectroscopy (MRS), Surface‐enhanced Raman spectroscopy (SERS), Tip‐enhanced Raman spectroscopy (TERS), Confocal Raman spectroscopy (CRS), and Scanning electron microscopy (SEM) were applied to confirm the correct synthesis and to investigate the function and properties of them in cells and tissues (Ashok & Canetta, 2020).

### 1.2 Nanomedicine

**FIGURE 1:**
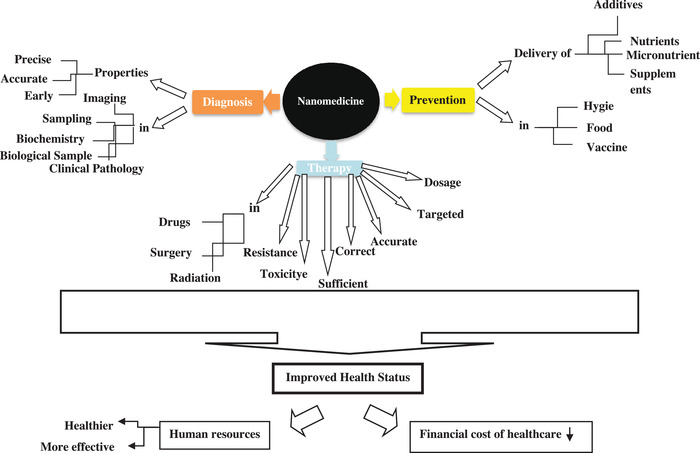
The different aspects of nanotechnology in medicine

One of the most important and useful fields in nanotechnology is the human‐health related section. In this case, the important point is to apply this technique and its products in different health fields, i.e., prevention (healthy food, purification of drinking water, and increased quality of soil and its fertilizer, nutrients, supplements, and vaccines), diagnostics (imaging and diagnostic systems in biochemistry and clinical histopathological laboratory), and therapeutics (drug, surgery, and radiotherapy) (Carvalhoa et al., 2020; Mohanty et al., 2014; Pati et al., 2009; Rajput, 2015; Raliya et al., 2016) [Figure 1, Swain et al., 2015; Uniyal et al., 2017; Xu et al., 2014; Zampoli Troncarelli et al., 2013].

**FIGURE 2:**
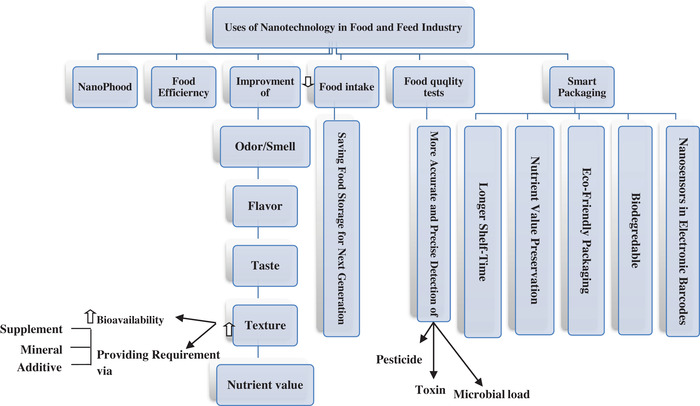
The use of nanotechnology in food and feed industry

In food industry, several advances are occurred due to nanotechnology and nanosensors which lead to boost food quality, nutrient value, bioavailability of mineral and vitamin especially for sensitive age‐groups, evaluation, checking, and monitoring of the food hygiene‐health in a case of free from pathogenic microorganism, poison, toxins, and food spoilage especially mycotoxicosis and packaging industry that the nanotechnology helps increasing food shelf life with the less nutritional loss and of course lowering the possible microbial load embraced novel nanotechnological tools (Aschberger et al., 2015; Cubadda, 2007; Hill & Li, 2017; Kumar, 2015; Martirosyan & Schneider, 2014; Omanovic‐Miklicanin & Maksimovic, 2016; Swain et al., 2016) [Figure 2]. Over‐the‐counter NPs in this field including titanium oxide (TiO_2_), zinc oxide (ZnO), Ag, colloidal metal, active nano iron (Fe)‐incorporated, complex nanoscale, nano‐sized nutrients, nano‐composite, silicate (Si), nanomicelle + glycerine, surface functional materials (Cubadda, 2007; Kumar, 2015; Martirosyan & Schneider, 2014). The dietary NP intake is estimated at about 10^12^ particles/day in the developed countries (Mohanty et al., 2014). Among these used particles, TiO_2_ and silicate have the highest usage; of course, different forms of NM have been consumed including NP, NT, NF, NM, liposome, and nanomembrane (Kumar, 2015; Martirosyan & Schneider, 2014).

There are several significant advances in pharmacology to produce drugs using new materials and formulas to act more effectively, precisely, and targeted (Mohanty et al., 2014; Shao et al., 2011; Zampoli Troncarelli et al., 2013; Shao et al., 2011). According to the high mortality rate of cancer, the antitumor drugs which act selectively with the lowest possible side effects on healthy cells are another interesting field (Mohanty et al., 2014). Obviously, the slow controlled release of these drugs is another aspect of nanotechnology. Another and very important application of nanotechnology is producing novel drugs that can overcome the resistance against the present drugs; the new antibacterial nano compounds are an example of this advantage (Hill & Li, 2017; Mohanty et al., 2014; Zampoli Troncarelli et al., 2013). In brief, more effective and specific drugs with the lowest possible dose and more efficient administration routes are the outcome of nanotechnology (Mohanty et al., 2014; Zampoli Troncarelli et al., 2013).

The reasons of the efficiencies of these new drugs are fundamental and even unexpected changes in materials due to their size decrement into nanoscale have been issued (Sauer, 2009; Swain et al., 2015; Zampoli Troncarelli et al., 2013). These obtained nano compounds have specific properties that include small size, higher bioavailability, bioactive chemical reactivity, surface solubility, stability of transformed material besides changes in their absorption, distribution, metabolism, excretion, and toxicology (ADMET) which followed Lipinski's rule of five (Kumar, 2015; Martirosyan & Schneider, 2014; Mohanty et al., 2014; Peran et al., 2012; Sauer, 2009; Zampoli Troncarelli et al., 2013).

Indeed, due to their size, availability to different cells, passage against biological barriers, and finally more absorption rates are addressed (Uniyal et al., 2017; Zampoli Troncarelli et al., 2013); furthermore, because of more surfaces, they act better (Swain et al., 2015; Zampoli Troncarelli et al., 2013). nanotechnology has not only been used in the anticancer and antibacterial industry but also there are several nanotechnological advances in vaccines, gene therapy, non‐steroid anti‐inflammatory drugs, hormones and endocrine, diabetic drugs, too (Martirosyan & Schneider, 2014; Sauer, 2009; Uniyal et al., 2017; Zampoli Troncarelli et al., 2013). In addition to the mentioned advantages of nanotechnology, there are other positive points such as combination therapy, decreased immunogenicity, drug toxicity, and stress of drug administration (Hill & Li, 2017; Martirosyan & Schneider, 2014; Mohanty et al., 2014; Sauer, 2009). Among mentioned fields, the most common ones can be a synthesis of antibacterial, antiseptic, cytotoxic, anticancer, wound healing, chemotherapy, brain drugs, wound dressing, and drug delivery NMs. The purpose of NP use in drug delivery is to increase level of drug in circulation, increased pay‐load, ability in drug solubility and sensitive molecule or drug protection against rapid degradation, immunity activity and endolysosomal degradation. polymerization in the presence of the drug in solution and drug adsorption by incubation in drug‐laden solution are two general methods for docking of NP and drug (Mitchell et al., 2021). different NMs have been used in pharmacology including ZnO NP, nanosilica (Si), magnetic NP, silicate oxide (SiO_2_) NP, Ag NP, metal NP, NT, fullerene, and solid lipid nanoparticles (SLNs) or lipid carriers, (Mohanty et al., 2014; Zampoli Troncarelli et al., 2013).

More nanotechnological studies are reported in engineering and medicine that are directly related to humans. The veterinary field is in the shadow of medicine and despite its present potentiality, the researches involving veterinary nanomedicine are scarce (Uniyal et al., 2017). In other words, although the essential needs for NMs have been sensed and its new techniques have been presented for several years; veterinary nanomedicine is in its infancy. The present status and possible potentials of nanotechnology in veterinary medicine namely “veterinary nanomedicine” will be discussed in this review (vide infra).

### 1.3 The benefits of nanotechnology in veterinary medicine

#### 1.3.1 Accurate, precise, complete, and safe prevention

The prevention is a rational strategy not only in medicine but also in veterinary medicine; because it inhibits the prevalence of infectious diseases and low profitability of hard‐resistant‐production diseases; furthermore, it results in shortening animal breeding cycle, more‐reliable products, and finally it decreases drug residues in these products as much as possible. Moreover, the criteria of carcass also have been improved in slaughter and market. All these aforementioned points affect human health and the environment.

Nanotechnology has been employed efficiently in preventive medicine. The economic aspect of nanotechnology is lowering the dose and amounts of antiseptics, disinfectants, and animal feed, even though it leads to higher efficiency which is translated as profit. The main principles in a successful prevention include proper nutrients, potentiation, and improvement of the immune system, diminution of the pathogenic microbial population as much as possible in the environment, food, and animal body, increased environmental hygiene of animal, and finally improved animal health.

Among new antimicrobial NMs, it can be christened Ag NP, NT, dendrimers, ZnO NP, and zinc (Zn) (Mohanty et al., 2014; Peran et al., 2012; Swain et al., 2016; Zampoli Troncarelli et al., 2013). The probiotics, ruminal microbial protein synthesis, decreased coliform content and intestinal pathogenic clostridiums versus increased useful microbial population such as lactobacillus and bifidobacteria which change ruminal fermentation and volatile fatty acid production; all of these mentioned points cause improved digestive health; consequently better and more efficient absorption of nutrients and improved health‐immunity and production (Gopi et al., 2017; Swain et al., 2015; Uniyal et al., 2017). The Ag NP, Zn N, and CNP Cu are materials used for the above‐mentioned aims (Gopi et al., 2017; Singh Sekhon, 2012; Uniyal et al., 2017).

Improved growth, nutrient bioavailability, feed efficiency, weight gain, and better physiological effects are caused by Zn NP, Ag NP, nano copper (Cu), nano Se, ZnO NP, and Fe NP (Gopi et al., 2017; Swain et al., 2015, 2016; Uniyal et al., 2017). The changes in blood biochemical profile have been reported due to the use of chromium (Cr) loaded chitosan NP (CNP), nano Cr, nano ZnO, nano Se, and nano Zn [Uniyal et al., 2017; Gopi et al., 2017; Hill & Li, 2017; Swain et al., 2016; Table 2].

**TABLE 2:** The changes in blood biochemical profile following intake of some nanomaterials

Blood biochemical parameters	
Decreased	Increased
ALT	ALP
LDL‐C	Serum Cr
TG	Serum TP
Cholesterol	HDL‐C
UN	Serum Lipase
Insulin	Total Blood, Serum, Tissue Se
Cortisol	
NEFA	

**ALP**: alkaline phosphatase, **ALT**: alkaline aminotransferase, **Cr**: chromium**, HDL**: high density lipoprotein cholesterol, **LDL**: low density lipoprotein cholesterol, **NEFA**: non‐esterified fatty acid, **Se**: selenium, **TG**: triacylglycerol, **TP**: total protein, **UN**: urea nitrogen.

**TABLE 3:** Applied nanoparticles in detection of infection diseases

Name	Major diagnostic applications
Florescent silica NPs (FSNPs)	Detection of infections with *Mycobacterium tuberculosis complex (MTB), Salmonella typhimurium, or S aureus*
Liposome‐based systems	Detection of food‐borne toxins such as cholera or botulinum toxins
Metal NPs known as surface plasmon resonance (SPR)	Detection of DNA/RNA sequences, or proteins
Single quantum dots‐based nanosensor	Detection of HIV‐1 and HIV‐2 viruses
Fluorescent immunosensor	Detection OF *Salmonella* (even at very low concentration), HVB, HCV, *Serratia marcescens* infection
NPs, NPs based magnetic resonance imaging(MRI) assay	Early diagnosis of acute renal failure (ARF) and renal ischemia

Another field in prevention is attained through improving immune profile, i.e., increased body Se content, serum antioxidant enzymes activity, plasma immunoglobulin (Ig) (IgA, IgM, IgG), γ‐globulins, superoxide dismutase (SOD), total globulin protein (TGP), catalase, glutathione peroxidase (GSHPX) besides decreasing pro‐inflammatory tumor necrosis factor‐alpha (TNFα) and DNA‐ fragmentation (Gamucci et al., 2014; Gopi et al., 2017; Uniyal et al., 2017). The above‐mentioned changes have been reported by using nano Cu, nano Se, nano Zn, and Zn NP (Gopi et al., 2017; Swain et al., 2015; Uniyal et al., 2017).

More interestingly, the advance in nanovaccines would be an important opportunity in preventive strategies in farm animals (Gill, 2013; Gopi et al., 2017; Sauer, 2009). The vaccine delivery and biodegradable nanosphere vaccine are two general applications of nanotechnology in the vaccine industry which result in better, safer, non‐infectious immune responses, and higher immunogenicity. They also can improve the solubility of hydrophobic antigens, have fewer side effects, sustainable control of antigens releases, target directly reticuloendothelial tissues or lymph nodes, and reduce the number of required doses and smaller volumes (Calderon‐Nieva et al., 2017; Mohanty et al., 2014; Rai et al., 2019; Sayed & Kamel, 2020; Zampoli Troncarelli et al., 2013). There is a report about the immunization of marines and foals against *Rhodococcusequi* following receiving vaccines containing NP adjuvant (Sauer, 2009).

The nanoantioxidants and nanoantiseptics are two other novel and developing fields in livestock or companion animal that improve the hygiene health of animals and the environment (Swain et al., 2015; Zampoli Troncarelli et al., 2013). All the above‐mentioned points in prophylaxis cause increased survival rate, health, longevity, and production; therefore the economic profits will increase the prevention of infection in the body besides no need for individual or herd eradication (Mohanty et al., 2014; Scott, 2005; Zampoli Troncarelli et al., 2013).

Another application of polymeric NPs is their use in gene and stem cell therapy instead of viral vectors. NPs can be applied as carried for transferring genetic materials. NPs vectors in comparison to other vectors are safer and allow loading with larger amounts of DNA. They have facile synthesis and flexible properties. These polymer NPs can be conjugated with genetic material via electrostatic attraction at physiological pH, thereby facilitating gene delivery (Rai et al., 2019; Sayed & Kamel, 2020).

## 2 DIAGNOSIS

Imaging is one of the most important arms of improved animal health in which several early malignancies and cancer cells can be determined in their precise position besides performing bioimaging (Mohanty et al., 2014). Therefore, several advances have been enough for more precise, safe, specific, and lower invasive imaging. NMs because of special properties such as better contrast, controlled biodistribution, and multi‐model imaging are developed as ideal imaging agents (Rai et al., 2019). The nano compounds employed in imaging include carbon nanotubes (CNT), fullerenes, polymeric nanospheres, dendrimers, nanoshells, SPIO_2_(MRI contrast), liposomes, magnetic NP such as FeO_2_ and polymeric NP like metal NP (Gill, 2013; Hill & Li, 2017; Mohanty et al., 2014; Singh Sekhon, 2012; Zampoli Troncarelli et al., 2013). Moreover, the invasive imaging by QD because of its physical properties such as high photostability and resistance to photobleaching has been used to improve understanding of animal gamete biology, oocyte movements, and cellular and molecular events during fertilization (Hill & Li, 2017; Rai et al., 2019). The nanosensors have been applied in searching for abortion reasons and real‐time evaluation of estradiol levels (Cubadda, 2007; Gopi et al., 2017; Kumar, 2015; Swain et al., 2016).

It is possible to diagnose cancer cells by tumor receptor detection(epidermal growth factor receptor, proepithelin), malfunction detection, location of tumor cells besides intercellular chemical analysis, and finding targeted cells with dendrimer, NT, nanoshells, iron oxide (FeO) NP and QD (Mohanty et al., 2014; Pati et al., 2009; Scott, 2005). Therefore, the NM and NP are effectively used in the diagnosis and detection of diseases; consequently, they can be helpful in rapid‐sensitive clinical diagnosis (Gopi et al., 2017; Hill & Li, 2017; Jain, 2007; Kumar, 2015; Mohanty et al., 2014; Sayed & Kamel, 2020). The obtained results greatly showed profitability and precise pathogen detection.

Nanotechnology also opened new apertures in laboratory diagnostics and the most important advances include QD, biochips, bioanalytical nanosensors, CNT, and information and communication technologies (ICT) (Omanovic‐Miklicanin & Maksimovic, 2016; Pati et al., 2009). These progressions enabled us to screen and detect protein, pathogens, toxins, heavy metals, and chemical agents in biological samples such as blood or tissues (Cubadda, 2007; Mohanty et al., 2014; Omanovic‐Miklicanin & Maksimovic, 2016; Scott, 2005). On the other hand, detective pathogenic tests of foods use NMs or nanotechniques to supply safer animal products for humans especially considering acute life‐threatening diseases like avian influenza or mad cow diseases (Omanovic‐Miklicanin & Maksimovic, 2016; Pati et al., 2009).

The “lab on a chip” as a molecular test is an NP‐based approach that cause less required sample, shorter run‐time, and real‐time data in the field (Hill & Li, 2017). For instance, the smart treatment delivery system detects animal disease and punctual treatment through salvia samples (Scott, 2005). The respirocytes nanosensor is another novel tool for detecting oxygen (O_2_)‐carbon dioxide (CO_2_) input‐output changes (Mohanty et al., 2014). Nanoparticles also can be applied as rapid, accurate, and effective methods for the detection of infectious diseases (Rai et al., 2019) (Table 3).

## 3 TREATMENT

The smarter mutant pathogens, different immune statuses in various animals, and their responses to pathogens have been considered three important animal life‐threatening risk factors and they cause diseases even if all the efforts have been tried for successful prevention and rapid, precise, punctual diagnoses. So, there is a vital necessity for a therapeutic action to be effective, specific, the right dose scheduled, least toxic, and with minimal residues in animal products. In this way, various applications of nanotechnology have been grown and according to the present reports, it also has several uses in veterinary medicine (Gopi et al., 2017).

antimicrobial drugs have been changed fundamentally by nanotechnology through producing new formulas or drug delivery systems. In this way, the required dose of antibiotics has been decreased as minimum as possible; moreover, other advantages include targeted therapy, slow‐controlled drug release, decreased drug toxicity, increased drug efficiency, and a solution for drug resistance (Hill & Li, 2017; Pati et al., 2009; Zampoli Troncarelli et al., 2013).

Some reports indicated synergism between antibiotics and NPs [e.g., 1]. This antimicrobial effect has been observed against bacteria, viruses, fungi, yeasts, and even African trypanosomes (Hill & Li, 2017; Mohanty et al., 2014; Zampoli Troncarelli et al., 2013). The development of NPs combined with antibodies or nucleic acid can be created rapid, sensitive, specific, and portable diagnostic assays (Alizadeh et al., 2013).

A wide range of bacteria including *E. coli, Moraxella, Brucella melitensis*, *Salmonella typhi, S. aureus, Mycobacterium, Anaplasma, Rhodococcus*, and *Ehlershia* are sensitive to nanocompounds (Bergin & Witzmann, 2013; Hill & Li, 2017; Ramachandraiah et al., 2015; Zampoli Troncarelli et al., 2013). In this line, animal diseases like bovine mastitis, tuberculosis, infectious bovine keratoconjunctivitis, trypanosomiasis, and evidently diarrhea can be treated by these nanocompounds (Swain et al., 2016; Zampoli Troncarelli et al., 2013).

Among NMs used as antimicrobial, Ag NP, ZnO NP, Zn nano, dendrimer, cylinder carbon C_60_, metal NP, polymeric NP, nanoemulsions are common instances (Fondevila, 2010; Hill & Li, 2017; Moen et al., 2009; Mohanty et al., 2014; Swain et al., 2016; Zampoli Troncarelli et al., 2013). In this continuum, liposomal amphotericin is an antifungal nano drug while nanopropolis is another high effective antimicrobial compound that reduces the required propolis versus its basic form (Bulbake et al., 2017; Olivia et al., 1995; Seven et al., 2018; Wang et al., 2012). The TiO_2_ NP dispersed in deionized water has been used as antimicrobial sprays and deodorants in pets (Zampoli Troncarelli et al., 2013).

The combination of tilmicosin and SLN has therapeutic potential in bovine mastitis (Ianiski et al., 2022; Zhu et al., 2018). The viruses also can be affected by NM; for example, the nanocrystals activated dendritic macrophage against the human immunodeficiency virus (HIV); furthermore, polysuolfonate G4 polyamidoamine (PAMAM) dendrimer blocked HIV activity (Gill, 2013; Zampoli Troncarelli et al., 2013).

ZnO and CuO can be efficient for the treatment of multiple drug resistance (MDR) pathogens, *E. coli, Streptococcus mutans*, and *Klebsiella pneumonia*. The NPs also can be applied to diminish the adverse effects of ischemia and can be used to treat many non‐infectious chronic metabolic diseases (Adibhesami et al., 2017; Rai et al., 2019).

The better and more advanced wound‐healing agents are another gift of nanotechnology in veterinary medicine (Hill & Li, 2017; Mohanty et al., 2014; Zampoli Troncarelli et al., 2013). In this regard, polymeric polyacrylate and Ag NP are two examples in this category (Hill & Li, 2017; Jaiswal et al., 2013; Mohanty et al., 2014; Zampoli Troncarelli et al., 2013). The other uses of nanodrugs include pain relief, increased O_2_ delivery in blood by respirocytes or increased phagocytosis potential and infectious clearing by using Microbivore (Mohanty et al., 2014; Van Saun, 2011).

The SLNs have several advantages in designing nanodrugs (Mohanty et al., 2014) as follows:

1. Passing brain‐blood‐barrier, therefore they can be used as central nervous system drugs
2. Induction of oral absorption through mucosal attachment
3. Slow‐controlled drug release
4. Increased drug functional efficiency
5. Variable administration routes [intramuscular, intravenously (IV), oral]

nanotechnology also makes a big change in different stages of the reproductive system to optimize the general reproductive performance and assisted to improve reproductive technology. The different NPs have been prescribed in this field include magnetic NPs, Si NP, nucleic acid, protein, polymeric [chitosan, polyethylenimine, poly(2‐dimethylamineo)ethyl methacrylate (PMMAEMA)]; it should be mentioned that the aims of these applications focused on increased fertility, diagnosis, and treatment of reproductive disorders, sustained release of reproductive hormones, the addition of effective compounds to extenders,spermatozoa preservation, sperm purification, sperm cryopreservation, sorting and freezing sperms, more success in female insemination, supporting female animals against possible infections, genetic engineering, longer and safer sperm transformation to other countries, increasing success in artificial insemination rate and consequently more economic profitability (Alizadeh et al., 2013; Hill & Li, 2017).

Some metallic NPs depending on the toxicity also can be applied for animal sterilization as contraceptives such as Cd (Alizadeh et al., 2013).

The polymeric nanosphere has been used in transdermal drug delivery (Zampoli Troncarelli et al., 2013). The NMs also have been used as carries for genes, molecules, and drugs with low solubility (Scott, 2005). These structures include Au NP, fullerene, NT, and polymeric micelles (Moen et al., 2009; Mohanty et al., 2014; Zampoli Troncarelli et al., 2013). The other benefit of NM is its application in releasing the main drug. For example, the combination of indomethacin and poly‐n‐vinyl pyrrolidone caused sustained release and decreased time‐interval use of drugs (Zampoli Troncarelli et al., 2013).

Cancer therapy is one of the most active fields in using nanodrugs. The use of NPs in cancer therapy creates suitable individualized therapeutic programs according to the type/stage of cancer, health condition, control dose of drug (Rai et al., 2019), predominance of drug resistance, etc. A huge number of studies focused on cancer therapy through drug delivery of chemotherapeutic drugs. The NMs in this field include Au NP, CNT, fullerene, liposomes, nanoshells, dendrimers, metal NPs, NTs, FeO NPs, QDs, and NPs derived from glucose or sucrose moieties. Oral administration, vaccination, and aerosol‐based drug delivery are common routes to enter of NPs in the body (Hill & Li, 2017; Mitchell et al., 2021; Mohanty et al., 2014; Pati et al., 2009; Scott, 2005; Seven et al., 2018; Zampoli Troncarelli et al., 2013).

It should be stated that all fields in nanodrugs were not discussed in this review. Generally, there are different reasons for choosing NMs in the drug industry like (Mohanty et al., 2014; Pati et al., 2009; Scott, 2005; Zampoli Troncarelli et al., 2013):

1. Lower use of active pharmaceutical ingredients
2. Decreased drug resistance owing to variable formula
3. Increased therapeutic efficiency through precise identification areas such as brain or intracellular media, increased drug bioavailability, and solubility
4. Combination therapy
5. Decreased present drug's toxicity and side effects
6. Different and even novel administration route
7. Increased drug lifetime
8. Slow‐controlled drug release
9. Decreased drug residue in animal and withdrawal time

### 3.1 Animal production

The main purpose of animal raisers is to reach the ideal slaughter weight, successful breeding, diminish diseases, and finally proper profit (Hill & Li, 2017). The food, nutrients, probiotics, functional foods, feed additives, feed supplements, antibiotics, and drugs have been used to shorten the production cycle and reach the mentioned goal (Van Saun, 2011). The uses of these compounds have their specific consequences such as drug resistance and drug residue in meat and other animal products (Zampoli Troncarelli et al., 2013). Using of nanominerals in the animal feed industry has helped to achieve these goals. They have various advantages such as cheaper, growth‐promoting, immunostimulating effects, regulation of the rumen fermentation process, needed in lower concentrations, and control of pathogens present in the feed (Alizadeh et al., 2013).

For example, antibiotics as one of the most important feed additives cause drug resistance or residual depots in animal products which NMs as mentioned before can be useful to decrease these disadvantages besides banning pathogen entrance into animal production (Hill & Li, 2017; Wang & Vermerris, 2016).

Trace minerals are another nanoform feed additive that have several benefits including better bioavailability, reduced dose, more stable reaction with other compounds, more nutritive value, increased production and weight gaining, improved growth, function, metabolism, digestibility of feed, and finally smarter immune system, increased distribution‐stability under high temperature and pressure‐ slaughter percentage‐half eviscerated‐ carcass lean percentage‐ muscle weight and decreased fat of carcass (Aschberger et al., 2015; Gopi et al., 2017; Hill & Li, 2017; Kumar, 2015; Martirosyan & Schneider, 2014; Swain et al., 2016; Uniyal et al., 2017).

**FIGURE 3:**
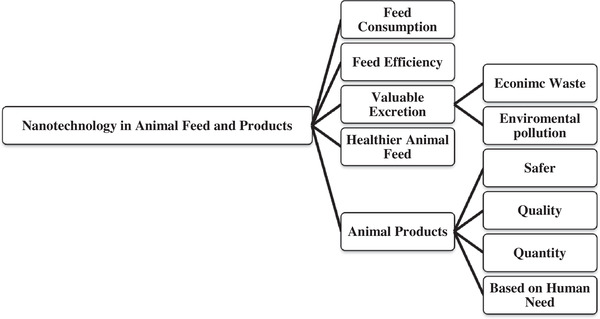
Usage of nanotechnology in animal feed and products

**FIGURE 4:**
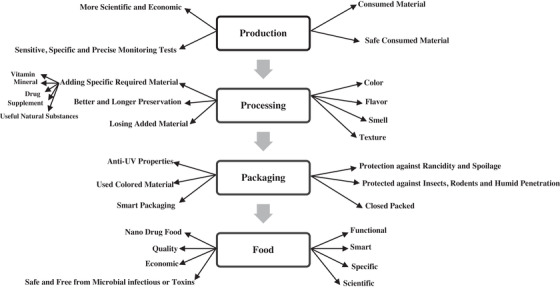
Food industry and nanotechnology

The food industry is one of the most principal target markets of nanotechnology (Kumar, 2015) [Figure 3]. The food industry uses different nanotechnological toolboxes to produce high quality, healthier feed, feed processing include food, drugs, improved color, texture, and flavor of foods and longer preservation of feed, reduced mycotoxicosis risk (MgO‐SiO2) and prevention of off‐taste due to oxidative reactions (Gopi et al., 2017; Kumar, 2015; Martirosyan & Schneider, 2014; Uniyal et al., 2017). Furthermore, nanotechnology improved packaging systems like conservation (nano‐zinc oxide), anti‐ultraviolet (UV) packaging (nano‐ TiO_2_), increased shelf life and maintenance time in stores, and nanosensors in an electronic barcode (for detection of any biological or chemical contamination in a very low concentration) (Inetianbor et al., 2015) [Figure 4, Kumar, 2015; Martirosyan & Schneider, 2014; Ramachandraiah et al., 2015].

Another use of nanotechnology in foods is transforming probiotics, nutritive, drugs, essential oils, omega 3 fatty acids, flavors, antioxidants, minerals, vitamins, phytochemicals, antimicrobials, and coenzyme Q_10_ to target tissues in specific diseases or for special purposes (Aschberger et al., 2015; Gopi et al., 2017; Hill & Li, 2017; Kumar, 2015; Mohanty et al., 2014; Uniyal et al., 2017). Another benefit of nanotechnology in foods includes widening the range between toxic and optimal dose, effective retention in the body, reduced nutritive excretion; therefore, decreased environmental pollution. Not only food production but also quality control can be improved by NM or nanosensors (Gopi et al., 2017; Martirosyan & Schneider, 2014; Uniyal et al., 2017). In other words, little amounts of poisons, toxins, pathogens, spoilage, and other harmful materials can be detected fast, precisely, and cheaper (Kumar, 2015; Mohanty et al., 2014; Omanovic‐Miklicanin & Maksimovic, 2016). The additives are extra materials added to food during its production or processing and are a component of food to improve its quality (Inetianbor et al., 2015; Ivani et al., 2012). The “on‐demand food” is a kind of food that retains latent in the body and delivers nutritive when the requirement is increased (Gopi et al., 2017). Besides, the additives and on‐demand supplements and contact materials are other related‐food fields that use nanotechnologies in different ways (Gopi et al., 2017; Inetianbor et al., 2015; Martirosyan & Schneider, 2014).

Naturally, NMs should be biodegradable and their bioaccumulation must be considered very carefully (Gopi et al., 2017; Hill & Li, 2017; Kumar, 2015). The improved nutritive quality of food products for human consumption such as increased milk calcium (Ca), vitamin D contents through using nanopowder shell or caseinate NP (Hill & Li, 2017; Pati et al., 2009). The different nanominerals, nanocarriers of bioactive compounds and NM such as polylactic‐co‐glycolic acid (PLGA)‐chitosan have positive effects on meat quality (Bergin & Witzmann, 2013; Hill & Li, 2017).

The nanoidentity preservation (IP)‐system in the meat industry is very effective because food issues become more important for people than past. Therefore, a system can be successful that gives the most details and specific information to the customers. The nano IP system provides data from origin farm, animal birth, animal history, and its activity, feed and welfare to slaughter and meat packaging factories (Pati et al., 2009; Scott, 2005). More specifically, this technology can be used in the poultry and aquaculture industry, i.e., their meat, eggs, breeders, feather, etc. This technology is helpful not only for customers but also for productive farm's owners in the quality of safety and security of animal products insurance (Pati et al., 2009).

A wide range of different NM shapes has been used in the food industry such as NPs, nanocapsules, nanovesicles, liposomes, NTs, micelles, nanoemulsions, nanofibers, nanocomposites, nanofilms, nanomembranes, nanochelates, nanocrystals, dendrimers, nanowires, and polymers (Hill & Li, 2017, 2017; Kumar, 2015; Martirosyan & Schneider, 2014). The three synthetic methods for nanominerals include physical, chemical, and biological ones. The different materials that are used in food fields (production, processing, smart packaging) include Ag, Si, TiO_2_, Au, Zn, ZnO, Mn, Fe, Cu, CNPCu, Cr, Se, CNT, magnesium (Mg), Ca, SiO_2_, Cd, Ti, Iridium (Ir) and Platinium (Pl) (Aschberger et al., 2015; Gopi et al., 2017; Hill & Li, 2017, 2017; Kumar, 2015; Martirosyan & Schneider, 2014; Omanovic‐Miklicanin & Maksimovic, 2016; Swain et al., 2015, 2016; Uniyal et al., 2017). Some of them have commercial uses such as nano‐ZnO which improves the growth rate, immune and reproductive status, decreases the prevalence of diarrhea, increases milk production, and reduces the somatic cell counts in cows (Rai et al., 2019).

The NMs can be effective in normal physiology and the function of body systems. The reproductive system is considered one of the most important systems for producers and is an opportunity for using nanotechnology in improving testis microstructure, semen quality, sperm purification, and sorting of normal spermatozoa; the examples of used NPs to follow mentioned aims are nanoantioxidants, Zn NP, Se NP, proper dosage of TiO2, polymeric NP and Si NP (Gopi et al., 2017; Hill & Li, 2017; Swain et al., 2015, 2016).

The gastrointestinal system is another important animal functional system. The NMs can affect absorption, balance level, and excretion of several elements through alteration of absorption and distribution. Moreover, these compounds have several effects on digestive microbial flora and manipulate their population resulting in using nutrition and drug compounds, more production from lower consumed materials, better growth, and decreased disease; Zn NP, CNP Cu, Cu nano, and Ag NP are classified as GI helper NPs (Gopi et al., 2017; Hill & Li, 2017; Swain et al., 2015; Uniyal et al., 2017).

The immune system, another important body system, influences animal health and its profitability. This system such as the other two mentioned ones also uses NMs to improve its functions like affecting Ig level, γ‐globulin, complement system, oxidative defense enzymes, and antioxidants. To reach these advantages, nano Se, Cr nanocomposite, nano Cu, and nano Zn have been studied (Gopi et al., 2017; Hill & Li, 2017; Sauer, 2009; Swain et al., 2016; Uniyal et al., 2017).

### 3.2 Disadvantages of nanotechnology

The NMs have their disadvantages besides their advantages. Certainly, specific positive criteria of NMs have negative aspects in some situations. The factors that influence the biotoxicological properties of engineered NMs (ENMs) include physiochemical characteristics and posologies (Martirosyan & Schneider, 2014). Toxicities of NP include passing through biological barriers (brain‐blood barrier (B.B.B.), blood, placenta, milk, and gut), increased biological availability, good distribution in body and organs, alteration of bioavailability of other nutrients, and deleterious effect on enzymes or proteins besides tissue accumulation, especially in bone, spleen, liver, and brain tissues (Cubadda, 2007; Gholamine et al., 2017; Gopi et al., 2017; Ivani et al., 2012; Martirosyan & Schneider, 2014). The other important issue is the potential accumulation of NMs and toxins in edible animal products like eggs or meats that can be harmful to human health, indirectly (Hill & Li, 2017; Zampoli Troncarelli et al., 2013). For example, the maximum AgNP accumulation is in the liver and spleen while CrNP accumulates particularly in the liver. The other NPs like Tio_2_ and Al silicate accumulated in intestinal lymph nodes (Hill & Li, 2017; Martirosyan & Schneider, 2014).

The increased reactive oxygen species activity in inflammatory digestive diseases has been reported following NP intake (Gopi et al., 2017). Furthermore, ulcerative colitis, Crohn's disease food allergy, and mucosal immunity failure have been observed following oral intake of ENMs; In this line, ENMs can be considered toxic to growth and the endocrine system through altering levels of antioxidants and sex hormones (Martirosyan & Schneider, 2014). The increased sperm death and reduced sperm viability have been observed following its incubation with NPs like ZnO and TiO_2_ specifically in high doses; In contrast to this report, increased sperm capacitation and final maturation stage were occurred after exposure to low dose TiO_2_ (Hill & Li, 2017).

The NP affects the reproductive system through oocyte cytotoxicity, genotoxicity, damaged oogenesis, follicle maturation, spermatogenesis, testis morphology, and gonadal tissue viability (Martirosyan & Schneider, 2014). In this context, ZnO NP showed cytotoxic effects via free radicals production, oxidative stress, lipid peroxidation, cellular membrane damage, and DNA oxidative damage (Swain et al., 2016). Regard to the present results, TiO_2_ can be carcinogenic and genotoxic, especially in sensitive groups (Bergin & Witzmann, 2013). TiO_2_ NP is a very stable and toxic NP that passes the barrier like BBB and blood‐placenta barrier besides its carcinogenic potential (Martirosyan & Schneider, 2014). The toxicity of poly (2‐dimethylamino) ethyl methacrylate (qpDMAEMA) through induction of hemolysis and sudden death has been reported following IV administration in mice (Hill & Li, 2017). Some reports indicate the development of autoimmune diseases like scleroderma, lupus erythematosus, and rheumatoid arthritis (RA) following NP exposure (Martirosyan & Schneider, 2014).

In essence, with regard to the limited NM toxicological studies and several unknown issues in this field (Bergin & Witzmann, 2013; Gopi et al., 2017; Hill & Li, 2017; Martirosyan & Schneider, 2014; Swain et al., 2016), there is an essential need to complete a database, especially in vivo research about pharmaco‐and toxico‐kinetics, pharmaco‐ and toxico‐dynamics, and even pharmaco‐ and toxico‐genomics of nanocompounds (Swain et al., 2016). Therefore, the risk assessment, regulatory policy, and oversight should be considered for using NP in animal feed (Gopi et al., 2017).

### 3.3 Prospective

nanotechnology has caused many advances in different fields. The various materials and modifications of pristine materials have been used in this avenue of research. The NMs have special characteristics including high strength weight ratio, higher stability, biodegradability, and biocompatibility. Different nanocomposite classifications include many structures and shapes‐ceramic, metal, polymer, glass, fiber, and particle. These materials have wide usages in many fields from engineering to medicine.

**FIGURE 5:**
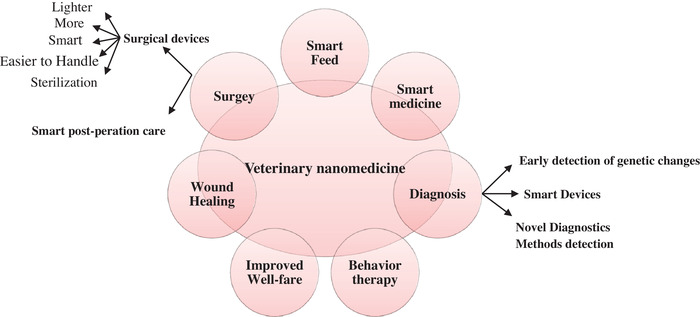
Nanotechnology prospective in veterinary medicine

The nanoindustry applications in veterinary medicine may have not become routine in professional tasks yet. Of course, according to the progressive world of NMs which originated from different materials in many shapes, the application fields of this technology are going to have many unknown pathways in the future [Figure 5]. This was a short summary in the growing world of biomedicals using nanocomposites particles. Scientists try more and more to recognize, discover, synthesize and develop eco‐friendly NMs. Natural modeling is used to improve the unity of nature, humans, and life. Although the research in this field is growing, its speed and scope have the potentials to be hastened.

## 4 CONCLUSIONS AND REMARKS

**FIGURE 6:**
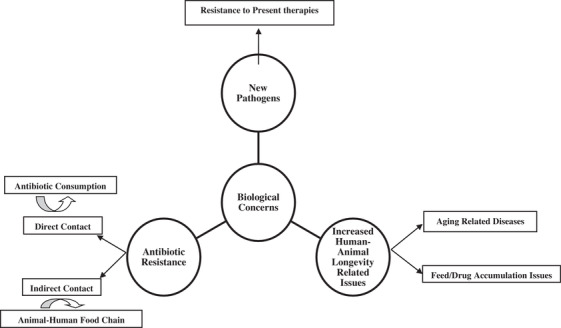
Nowadays biological concerns

Many reasons determine the necessity for employing novel, more effective, and safer technology and materials. These reasons include fading present materials and fuel resources, surging environmental pollution, and world concern phenomena, i.e., global warming, besides several biological issues [Figure 6]. Nanotechnology is a novel discipline that led to noticeable advances in biomedical engineering. Indeed, nanotechnology was presented by famous physicist, Richard Feyman, in order to integrate basic sciences such as chemistry, physics, material science, electronics, biology, and medicine (Khan et al., 2019). This technology introduced new used compounds to the modern world by the manipulations and use of the nano‐particles, 1–100 nm, although the particle size of nano‐carriers in the biomedical field has been even defined up to 1000 nm (Scott, 2005). Generally, this technology has been used in various medical fields such as regenerative medicine, tissue engineering, cell therapy, imaging, biomarker detection, drug and gene delivery besides treatment of cancer, and cardiovascular, orthopedic, and neurological diseases. The usage of nanotechnology in veterinary medicine (veterinary nanomedicine) is still in its infancy. These applications bring many advantages such as efficient materials for producing and breeding animals, novel, safer and more efficient preventive diagnoses and more economic therapies for animals and improving animal welfare terminating to more‐better and safer production for human consumption with higher economical profitability. There is an urgent need for more research addressing NM pharmaco‐ or toxico‐kinetics in various farm animals, allergic and toxic reactions because of heterogeneous materials and their shapes, formula, and doses, animal species, and other unknown issues. On the other hand, these materials have their disadvantages that originate from positive structural changes in the targeted field. One of the most important points is to evaluate and compare safety between a high‐risk substance with lower exposure and a limited risky substance with wide exposure (Gopi et al., 2017).

In sum, it is necessary to perform wide and cohort studies about the NMs, their effects, their posology for different purposes, determination of their toxicities, and possible impacts on animal products due to entrance into the human food chain. Regard to changes in nowadays lifestyle, modern and new technology are an inseparable part of the human future. So, it seems that veterinary nanomedicine has a bright future ahead.

## AUTHOR CONTRIBUTIONS

Fariba jafary: Data collection and analysis. Shima Motamedi: Data collection and analysis. Isaac Karimi: Content approval and information.

## CONFLICT OF INTEREST

The authors declare that they have no conflict of interest.

### PEER REVIEW

The peer review history for this article is available at https://publons.com/publon/10.1002/vms3.1050

## Data Availability

Data sharing not applicable to this article as no datasets were generated or analyzed during this study.
